# Predictive efficacy of neutrophil-to-lymphocyte ratio for long-term prognosis in new onset acute coronary syndrome: a retrospective cohort study

**DOI:** 10.1186/s12872-020-01773-x

**Published:** 2020-11-30

**Authors:** Yi Yang, Yanan Xu, Jun Wang, Xueqin Zhai, Haibing Jiang

**Affiliations:** 1 Department of Cardiology Fourth Ward, The Xinjiang Medical University Affiliated Hospital of Traditional Chinese Medicine, Urumqi, 830011 China; 2 The People’s Hospital of Xuancheng City, Anhui, 242000 China

**Keywords:** Acute coronary syndrome, Gensini score, Neutrophil and lymphocyte ratio, Major adverse cardiovascular events

## Abstract

**Background:**

Inflammation is involved in the pathogenesis and progression of coronary artery diseases (CADs), including acute coronary syndrome. The neutrophil-to-lymphocyte ratio (NLR) has been identified as a novel marker of the pro-inflammatory state. We aimed to evaluate the predictive efficacy of the NLR for the prognosis of patients with new-onset ACS.

**Methods:**

We retrospectively included consecutive patients with new-onset ACS treated with emergency coronary angiography. NLR was measured at baseline and analyzed by tertiles. The severity of coronary lesions was evaluated by the Gensini score. Correlations of NLR with the severity of CAD and the incidence of major adverse cardiovascular diseases (MACEs) during follow-up were determined.

**Results:**

Overall, 737 patients were included. The NLR was positively correlated with the severity of coronary lesions as assessed by Gensini score (*P* < 0.05). During the follow-up period (mean, 43.49 ± 23.97 months), 65 MACEs occurred. No significant association was detected between baseline NLR and the risk of MACEs during follow-up by either Kaplan–Meier or Cox regression analysis. Multivariable logistic regression analysis showed that a higher NLR was independently associated with coronary lesion severity as measured by the Gensini score (1st tertile vs. 3rd tertile hazard ratio [HR]: 0.527, *P* < 0.001, and 2nd tertile vs. 3rd tertile HR: 0.474, *P* = 0.025).

**Conclusions:**

The NLR may be associated with coronary disease severity at baseline but is not associated with adverse outcomes in patients with new-onset ACS.

**Ethics Approval Number:**

2019XE0208

## Background

The current understanding of the pathogenesis of atherosclerosis is focused on the "inflammatory hypothesis of atherothrombosis" theory [1, 2]. Inflammatory cells and inflammatory signaling pathways play complex roles in the process of atherosclerosis, including initiating repair after vascular injury and mediating plaque instability and rupture, finally leading to acute coronary events [3–6]. Patients with acute coronary syndrome (ACS), particularly those with new-onset ACS, often have an unstable clinical status and a poor prognosis, and optimization of risk stratification is clinically important in this patient group [7, 8].

Pathological studies have confirmed an increase in white blood cell mobilization in necrotic areas of the myocardium [9]. Moreover, white blood cell count, a clinical marker of universal inflammation, was shown to be independently associated with the risk of mortality and incidence of major adverse cardiovascular events (MACEs) in ACS patients [10, 11]. However, white blood cell count is unstable and tends to be affected by comorbidities such as infection. Interesting, it has also been indicated that decreased lymphocyte numbers may be associated with acute coronary events [12]. Recent studies showed that the neutrophil-to-lymphocyte ratio (NLR), which incorporates two major subgroups of white blood cells, may confer prognostic efficacy in many diseases, including inflammatory diseases, cardiovascular diseases, and malignancies [13, 14]. It has been suggested that an elevated NLR is associated with increased long-term mortality in patients with acute myocardial infarction (AMI) complicated by left main-and/or three-vessel disease [15]. Moreover, the role of the NLR for the management of patients with coronary artery disease (CAD) has also been evaluated, and the results showed that the NLR is correlated with CAD severity [16–18]. However, these studies were of limited scale and patients with a previous diagnosis of CAD were not excluded. Overall, clinical and experimental data support an important role for inflammation in CAD [1, 2].

Whether the NLR remains a significant prognostic factor after control for the severity of coronary lesions in new-onset ACS remains to be determined. Therefore, in this study, we retrospectively enrolled patients with new-onset ACS to comprehensively evaluate the potential prognostic role of the NLR in these patients.

## Methods

### Patients and study design

Consecutive patients with a first diagnosis of ACS who were admitted to the Xinjiang Uygur Autonomous Region Traditional Chinese Medicine Hospital affiliated to the Xinjiang Medical University from January 2011 to January 2019 were included. ACS was diagnosed in accordance with previously established guidelines [19]. Patients with the following clinical conditions that may affect the NLR were excluded: hepatic or renal dysfunction, malignant tumors, acute infection, connective tissue disease, physical and chemical damage, previously proven systemic inflammatory disease, and recent surgery. Moreover, patients with a previous diagnosis of CAD were also excluded. The protocol of the study was approved by the ethics committee of our local institution before enrollment of the patients. Informed patient consent was not needed due to the retrospective design of the study.

### Blood sampling and definitions of CAD risk factors

Venous blood samples were taken when patients initially presented to the emergency department or prior to angiography, and the samples were sent immediately for laboratory analysis. Hypertension was defined if the patient was taking any antihypertensive medications or had blood pressure measurements over 140/90 mmHg on least two separate occasions [20]. Diabetes was diagnosed based on medical history or measurements of fasting and/or postprandial glucose according to previous guidelines [21]. The estimated glomerular filtration rate (eGFR) was calculated with the Modification of Diet in Renal Diseases equation [22].

### Coronary angiography and Gensini score

All patients underwent coronary angiography within 12 h of admission. Two independent investigators assessed the degrees of stenosis of the coronary lesions. Consensus with a third investigator was indicated if disagreement occurred. The Gensini score (GS), which incorporates both the extent of luminal narrowing and the geographic importance of the lesion, was calculated to reflect the severity of coronary lesions [23]. We used the GS instead of the SYNTAX system to reflect the severity of coronary lesions, because the calculation method of SYNTAX integral is more complicated. This limits its use in clinical practice and makes it difficult to apply to emergency patients, such as those with new-onset ACS. Moreover, research has shown that the SYNTAX score cannot be utilized to define future risk as the Gensini score can in patients with non-obstructive CAD [24].

### Outcomes

Patients were followed by telephone interview or clinic visit. The primary outcome was all-cause mortality. The secondary outcome was a composite of MACEs, including cardiac mortality, non-fatal myocardial infarction and stroke, stent thrombosis, and revascularization (unplanned repeat PCI).

### Statistical analysis

Continuous variables were expressed as mean and standard deviation (SD) or median and interquartile range (IQR), whereas categorical variables were presented as percentages. Patients were grouped according the tertiles of the NLR or GS. One-way analysis of variance (ANOVA) was used to evaluate the difference in normally distributed numeric variables among the groups, while for the non-normally distributed variables, Mann–Whitney U test or Kruskal–Wallis variance analysis was used. For the categorical variables, a chi-square (χ^2^) test was employed. Linear regression analysis was performed to identify the factors associated with the GS. Prognostic factors for the occurrence of mortality and MACEs were analyzed with Kaplan–Meier survival method. Univariate analysis was first performed, and then significant variables were included in the multivariate Cox analysis. A *P* value < 0.05 indicated a statistically significant difference. All analyses were performed using SPSS 22.0 (SPSS Inc, Chicago, IL, USA).

## Results

### Characteristics of patients according to NLR

**Fig. 1:**
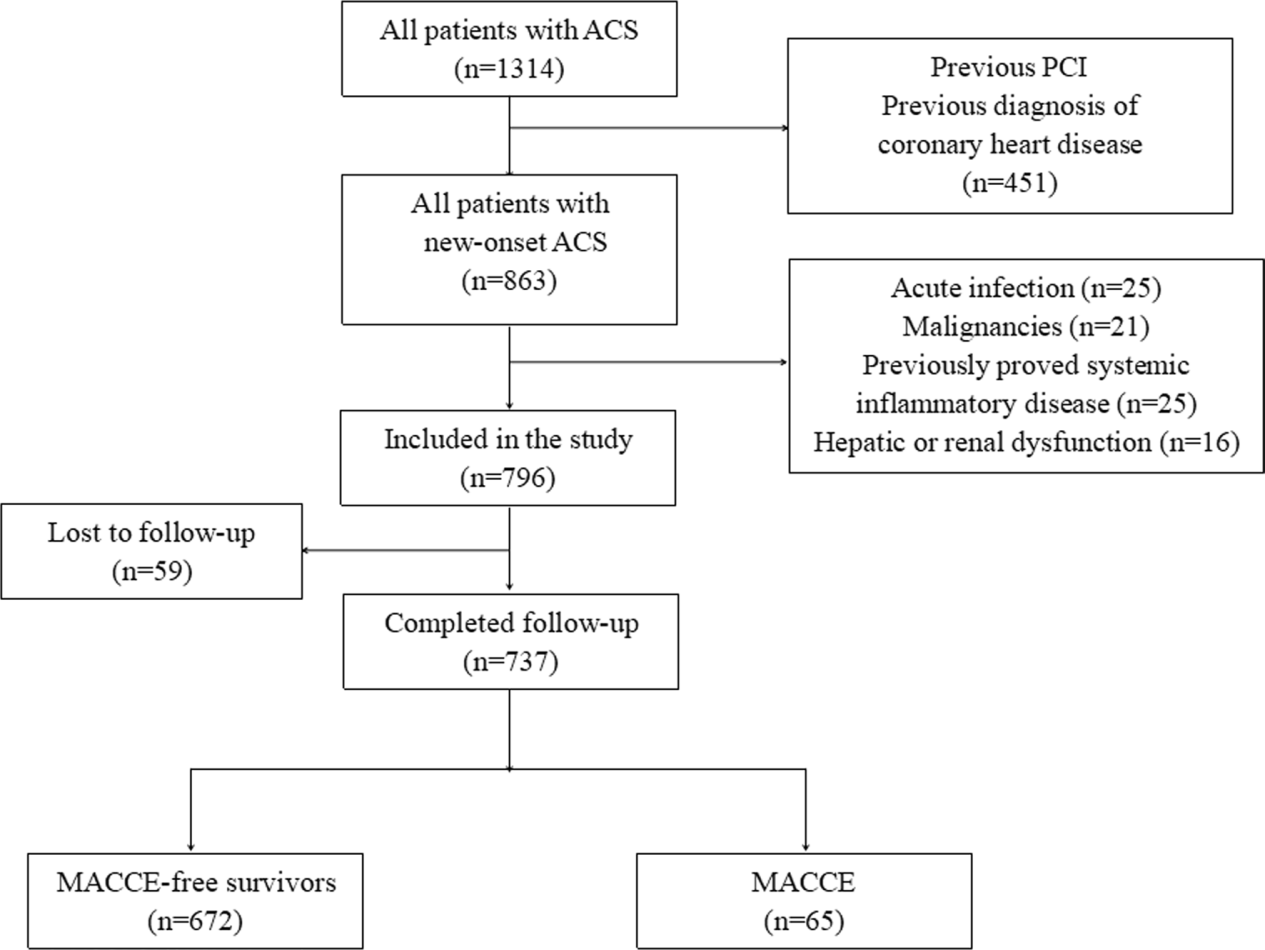
Flowchart of patient enrollment

A flow chart outlining patient enrollment is shown in Fig. 1. A total of 737 patients with new on-set ACS were included. The baseline characteristics of the included patients according to the tertiles of NLR are shown in Table 1. The results showed that patients with a higher NLR were more likely to have dyslipidemia and ST-elevation myocardial infarction (STEMI; both *P* < 0.05).

**Table 1:** Baseline characteristics of the included patients according to NLR tertiles

	1st tertile ≤ 3.37 (n = 245)	2nd tertile 3.38–6.93 (n = 245)	3rd tertile ≥ 6.94 (n = 247)	t/Z/χ^2^	P
Sex (male/female)	200/45	203/42	213/34	2.039	0.361
Age (years)	58.20 ± 12.46	57.20 ± 12.45	58.64 ± 12.04	0.877	0.417
Hypertension	113 (46.1)	115 (46.9)	103 (41.7)	1.582	0.453
Diabetes mellitus	76 (31.0)	64 (26.1)	53 (21.5)	5.820	0.054
DM treatment				3.808	0.433
Diet only	4 (6.6)	2 (3.6)	2 (4.9)		
Oral hypoglycemic drugs	23 (37.7)	30 (54.5)	21 (51.2)		
Insulin	34 (55.7)	23 (41.8)	18 (43.9)		
Smoking				9.319	0.054
Never smoker	110 (44.9)	132 (53.9)	115 (46.6)		
Former smoker	19 (7.8)	7 (2.9)	11 (4.5)		
Current smoker	116 (47.3)	106 (43.3)	121 (49.0)		
Alcohol drinking				2.390	0.664
Never drinking	151 (61.9)	149 (61.3)	146 (59.3)		
Former drinking	35 (14.3)	35 (14.4)	46 (18.7)		
Current drinking	58 (23.8)	59 (24.3)	54 (22.0)		
Family history of CAD	99 (40.4)	101 (41.2)	97 (39.3)	0.197	0.906
SBP (mmHg)	122.02 ± 20.97	122.91 ± 19.32	122.21 ± 19.17	0.137	0.872
DBP (mmHg)	75.96 ± 13.59	76.75 ± 12.90	77.23 ± 13.84	0.567	0.567
Heart rate (bpm)	82.78 ± 15.31	80.54 ± 14.71	82.50 ± 15.03	1.618	0.199
BMI (kg/m^2^)	25.11 ± 5.28	25.04 ± 5.15	24.63 ± 5.94	0.475	0.622
HDL-C (mmol/l)	0.95 ± 0.26	1.00 ± 0.26	1.01 ± 0.26	2.575	0.077
LDL-C (mmol/l)	2.93 ± 0.91	2.96 ± 0.84	2.97 ± 0.85	0.095	0.910
TC (mmol/l)	4.61 ± 1.22	4.66 ± 1.26	4.59 ± 1.08	0.181	0.834
TG (mmol/l)	1.76 ± 1.13	2.04 ± 1.06^a^	2.26 ± 1.17^ab^	11.988	< 0.001
ApoA1 (g/L)	1.21 ± 0.29	1.28 ± 0.53	1.22 ± 0.27	1.604	0.202
ApoB (g/L)	0.92 ± 0.49	0.90 ± 0.27	0.91 ± 0.26	0.160	0.852
Lp (a) (g/L)	244.88 ± 258.72	236.56 ± 227	239.63 ± 214.2	0.059	0.942
Creatinine (mmol/L)	79.27 ± 36.5	81.75 ± 56.31	79.25 ± 35.77	0.259	0.772
BUN (mmol/l)	5.42 ± 2.76	5.69 ± 2.26	5.68 ± 2.81	0.833	0.435
Uric acid (mmol/L)	334.27 ± 90.96	326.4 ± 88.92	325.73 ± 99.45	0.629	0.534
WBC	9.03 ± 3.21	11.05 ± 3.35^a^	12.32 ± 3.41^ab^	61.212	< 0.001
Monocyte count	0.63 ± 0.24	0.66 ± 0.34	0.46 ± 0.32^ab^	30.768	< 0.001
PLR	89.94 ± 36.52	145.34 ± 110.94^a^	265.13 ± 175.65^ab^	132.705	< 0.001
RBC	4.77 ± 0.72	4.85 ± 0.58	4.65 ± 0.82^b^	4.852	0.008
HGB	145.04 ± 22.16	144.99 ± 26.14	142.53 ± 28.78	0.759	0.468
PLT	224.49 ± 71.94	229.48 ± 144.24	236.26 ± 162.19	0.492	0.612
MPV	10.16 ± 1.43	10.26 ± 1.24	10.14 ± 1.66	0.499	0.608
PDW	13.36 ± 3.83	13.43 ± 3.71	13.31 ± 3.22	0.065	0.937
PCT	0.24 ± 0.08	0.23 ± 0.09	0.25 ± 0.12	1.632	0.196
Clinical diagnosis				20.160	< 0.001
UA	52 (21.2)	29 (11.8)	19 (7.7)		
NSTEMI	26 (10.7)	30 (12.2)	31 (12.6)		
STEMI	167 (68.2)	186 (75.9)	197 (79.8)		
Coronary artery lesion					
UPLMT	22 (9.2)	25 (10.6)	21 (8.7)	0.526	0.769
LAD	217 (88.9)	211 (86.5)	216 (87.8)	0.687	0.709
LCX	155 (64.3)	147 (61.0)	155 (63.0)	0.577	0.749
RCA	179 (74.0)	178 (73.3)	186 (75.3)	0.278	0.870
Medication situation					
Aspirin	20 (8.2)	32 (13.1)	22 (8.9)	3.782	0.151
Statins	18 (7.3)	28 (11.4)	21 (8.5)	2.625	0.269
β-Blockers	16 (6.5)	16 (6.5)	12 (4.9)	0.818	0.664
ACEI/ARB	8 (3.3)	13 (5.3)	6 (2.4)	3.049	0.218
CCB	41 (16.7)	26 (10.6)	35 (14.2)	3.885	0.143

^a^*P* < 0.05 compared to the 1st tertile; ^b^*P* < 0.05 compared to the 2nd tertile

### Incidence of mortality and MACEs according to the NLR

The incidences of clinical outcomes during follow-up (mean, 43.49 ± 23.97 months) for the included patients with new-onset ACS according to the NLR are shown in Table 2. No significant differences in the incidences of all-cause mortality, overall and components of MACEs, or bleeding events were detected among the three groups (all P ≥ 0.05).

**Table 2:** Incidence of adverse outcomes in ACS patients according to the NLR tertiles

	1st tertile ≤ 3.37 (n = 245)	2nd tertile 3.38–6.93 (n = 245)	3rd tertile ≥ 6.94 (n = 247)	χ^2^	P
ACM, n (%)	5 (2.0)	12 (4.9)	7 (2.8)	3.385	0.184
Non-sudden cardiac death, n (%)	1 (0.4)	0 (0.0)	0 (0.0)	1.825	0.665
MACE, n (%)	21 (8.6)	24 (9.8)	20 (8.1)	0.469	0.791
CM, n (%)	4 (1.6)	12 (4.9)	7 (2.8)	4.421	0.110
Re-myocardial infarction, n (%)	4 (1.6)	0 (0.0)	2 (0.8)	5.578	0.061
ST, n (%)	0 (0.0)	0 (0.0)	2 (0.8)	2.617	0.332
Revascularization, n (%)	11 (4.5)	17 (6.9)	12 (4.9)	1.666	0.435
Stroke, n (%)	1 (0.4)	0 (0.0)	2 (0.8)	2.764	0.251
Bleeding events, n (%)	1 (0.4)	6 (2.4)	1 (0.4)	5.904	0.052

### Characteristics of patients according to GS

The baseline characteristics of patients according to the tertiles of GS (1st tertile GS < 49; n = 250, 2nd tertile GS: 49 ~ 85; n = 246, and 3rd tertile GS > 85; n = 241) are shown in Table 3. The percentage of male patients, age, prevalence of diabetes mellitus, and history of smoking differed significantly among the groups according to GS tertile (all *P* < 0.05). However, we found no relationship between other indicators and coronary severity (all *P* > 0.05).

**Table 3:** Baseline characteristics of the included patients according to the GS tertiles

	1st tertile ≤ 45 (n = 250)	2nd tertile 49–85 (n = 246)	3rd tertile ≥ 85 (n = 241)	t/Z/χ^2^	P
Sex (male/female)	171/79	191/55	200/41	14.814	0.001
Age (years)	55.37 ± 12.26	58.23 ± 11.84^a^	60.53 ± 12.34^ab^	11.111	< 0.001
Hypertension	106 (42.4)	111 (45.1)	114 (47.3)	1.199	0.549
Diabetes mellitus	45 (18.0)	66 (26.8)	82 (34.0)^ab^	16.381	< 0.001
Diabetes mellitus treatment				0.846	0.932
Diet only	2 (4.2)	3 (6.7)	3 (4.7)		
Oral hypoglycemic drugs	23 (47.9)	19 (42.2)	32 (50.0)		
Insulin	23 (47.9)	23 (51.1)	29 (45.3)		
Smoking				9.963	0.041
Never smoker	134 (53.6)	119 (48.4)	104 (43.2)		
Former smoker	16 (6.4)	13 (5.3)	8 (3.3)		
Current smoker	100 (40.0)	114 (46.3)	129 (53.5)		
Alcohol drinking				1.053	0.902
Never drinking	147 (58.8)	153 (63.2)	146 (60.6)		
Former drinking	41 (16.4)	36 (14.9)	39 (16.2)		
Current drinking	62 (24.8)	53 (21.9)	56 (23.2)		
Family history of CAD	95 (38.0)	100 (40.7)	102 (42.3)	0.972	0.615
SBP (mmHg)	123.72 ± 20.64	122.37 ± 18.87	121 ± 19.85	1.157	0.315
DBP (mmHg)	76.86 ± 13.88	77.45 ± 13.12	75.62 ± 13.3	1.174	0.310
Heart rate	80.42 ± 14.05	81.78 ± 14.26	83.68 ± 16.58	2.930	0.054
BMI (kg/m^2^)	24.78 ± 5.95	24.9 ± 5.05	25.11 ± 5.36	0.203	0.817
HDL-C (mmol/l)	0.96 ± 0.23	0.99 ± 0.26	1.01 ± 0.28	2.152	0.117
LDL-C (mmol/l)	2.88 ± 0.85	2.94 ± 0.74	3.04 ± 1	1.434	0.239
TC (mmol/l)	4.63 ± 1.23	4.57 ± 0.98	4.68 ± 1.34	0.374	0.688
TG (mmol/l)	2.14 ± 2.15	2.15 ± 2.13	1.98 ± 1.45	0.426	0.653
ApoA1 (g/L)	1.2 ± 0.26	1.23 ± 0.3	1.29 ± 0.53	2.710	0.067
ApoB (g/L)	0.9 ± 0.27	0.88 ± 0.23	0.96 ± 0.52	2.433	0.089
Lp(a) (g/L)	234.48 ± 232.84	263.47 ± 280.29	222 ± 172.39	1.505	0.223
Cr (mmol/L)	80.9 ± 35.25	80.52 ± 42.9	78.84 ± 52.29	0.150	0.860
BUN (mmol/l)	5.71 ± 2.91	5.59 ± 2.87	5.5 ± 1.95	0.394	0.674
Uric acid (μmol/L)	326.22 ± 92.64	335.89 ± 94.27	324.19 ± 92.59	1.090	0.337
WBC	10.88 ± 3.74	10.87 ± 3.71	10.64 ± 3.29	0.339	0.712
Neutrophil count	8.13 ± 3.63	8.37 ± 3.58	8.11 ± 3.15	0.419	0.658
lymphocyte count	1.95 ± 1.27	1.76 ± 0.96	1.81 ± 1.07	1.954	0.142
NLR	5.24 ± 3.90	6.41 ± 5.24	7.46 ± 5.51	12.506	< 0.001
NLR tertiles				19.287	0.001
1st tertile	104 (41.6)	77 (31.3)	64 (26.6)		
2nd tertile	84 (33.6)	84 (34.1)	77 (32.0)		
3rd tertile	62 (24.8)	85 (34.6)	100 (41.5)		
Monocyte count	0.59 ± 0.32	0.58 ± 0.32	0.57 ± 0.31	0.258	0.773
PLR	158.09 ± 122.37	176.51 ± 159.31	166.74 ± 142.57	1.042	0.353
RBC	4.75 ± 0.70	4.74 ± 0.73	4.79 ± 0.72	0.399	0.672
HGB	145.15 ± 24.97	142.77 ± 27.85	144.62 ± 24.6	0.579	0.561
PLT	223.74 ± 69.67	242.01 ± 203.23	224.52 ± 77.66	1.508	0.222
MPV	10.20 ± 1.29	10.16 ± 1.75	10.2 ± 1.28	0.065	0.937
PDW	13.66 ± 3.35	13.44 ± 3.78	12.98 ± 3.63	2.298	0.101
PCT	0.24 ± 0.08	0.24 ± 0.11	0.24 ± 0.10	0.239	0.788
Clinical diagnosis				1.672	0.796
UA	38 (15.2)	32 (13.0)	30 (12.4)		
NSTEMI	32 (12.8)	26 (10.6)	29 (12.0)		
STEMI	180 (72.0)	188 (76.4)	182 (75.5)		
Aspirin	18 (7.2)	23 (9.3)	33 (13.7)	5.923	0.052
Statins	17 (6.8)	19 (7.7)	31 (12.9)^ab^	6.029	0.043
β-Blockers	15 (6.0)	12 (4.9)	17 (7.1)	1.027	0.598
ACEI/ARB	7 (2.8)	8 (3.3)	12 (5.0)	1.828	0.401
CCB	32 (12.8)	34 (13.8)	36 (14.9)	0.470	0.790

Abbreviations are as in Table 1

### Factors associated with coronary lesion severity as detected by Gensini Score

The results of multivariable logistic regression analysis showed that a higher NLR was independently associated with coronary lesion severity as measured by the GS (1st tertile vs. 3rd tertile hazard ratio [HR]: 0.527, *P* < 0.001, and 2nd tertile vs. 3rd tertile HR: 0.474, *P* = 0.025). The other factors independently related to GS included advanced age (HR: 1.033, *P* < 0.001), male gender (HR: 1.835, *P* < 0.001), and the absence of diabetes (HR: 0.507, *P* < 0.001; Table 4).

**Table 4:** Factors independently correlated with the severity of coronary arterial atherosclerosis as detected by GS: multivariate logistic regression analysis

Variables	B	SE	Wald	P	HR	95% CI	
						Lower limit	Upper limit
Age	0.032	0.006	27.046	< 0.001	1.033	1.020	1.045
Sex (male vs female)	0.607	0.173	12.363	< 0.001	1.835	1.309	2.575
Diabetes mellitus (No vs Yes)	− 0.680	0.163	17.508	< 0.001	0.507	0.368	0.696
Smoking							
Never smoker vs current drinking	− 0.178	0.152	2.501	0.079	0.837	0.216	1.255
Former smoker vs current drinking	− 0.237	0.171	3.390	0.059	0.790	0.310	1.098
Statins	− 0.470	0.245	3.666	0.056	0.625	0.386	1.011
NLR group							
1st tertile vs 3rd tertile	− 0.640	0.174	13.526	< 0.001	0.527	0.375	0.742
2nd tertile vs 3rd tertile	− 0.747	0.334	4.993	0.025	0.474	0.246	0.912

CI, confidence interval; HR, hazard ratio

### Predictors of clinical outcomes

**Fig. 2:**
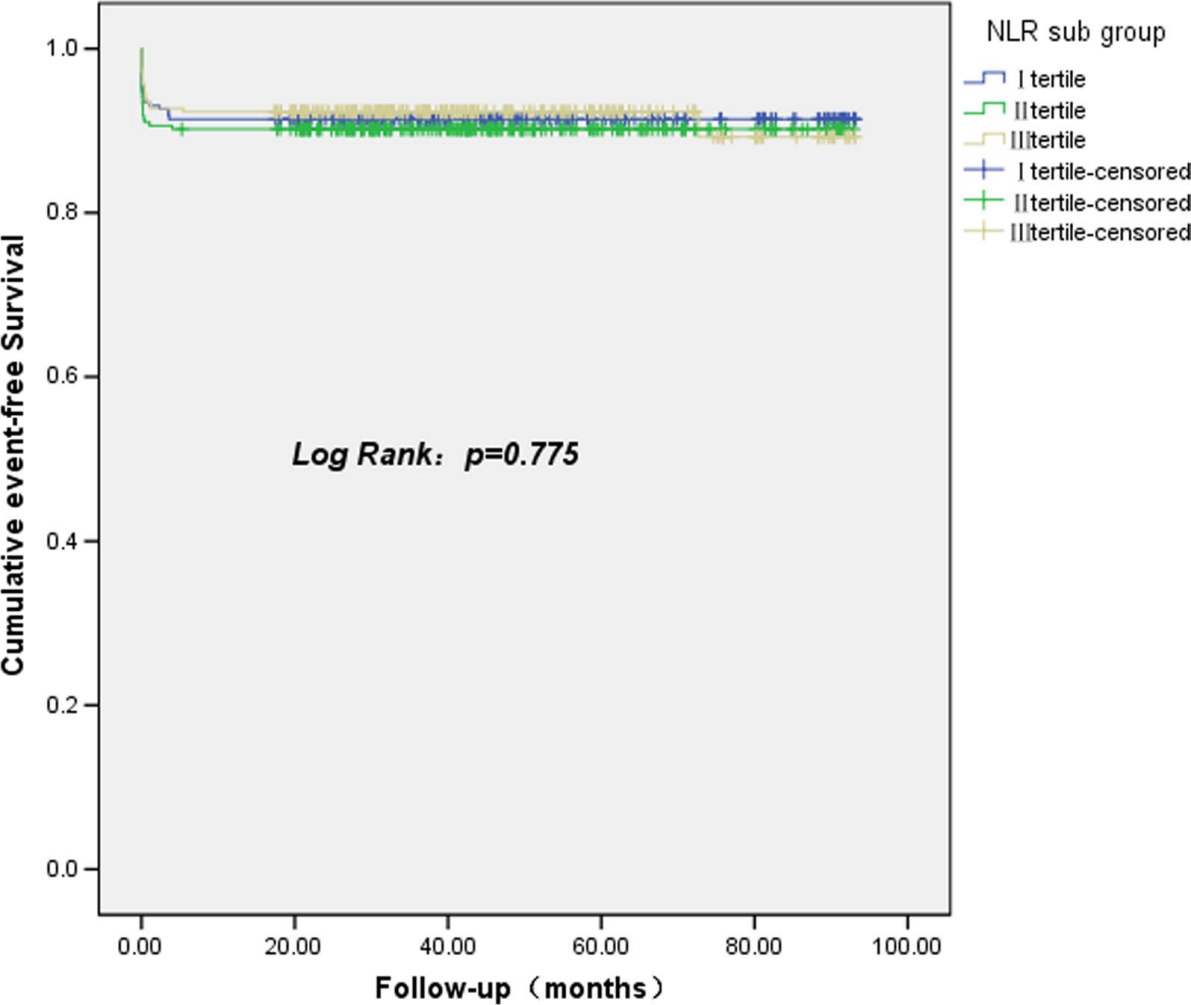
Cumulative event-free survival analysis according to NLR tertiles

Overall, 65 patients experienced MACEs during follow-up, including 23 (35.38%) cases of cardiac mortality, 6 (9.23%) cases of nonfatal MI, 2 (3.08%) cases of ST, 33 (50.77%) cases of revascularization, and three (4.62%) cases of nonfatal stroke. The NLR was not correlated with MACEs either as a continuous variable or according to tertiles (both *P* > 0.05). Kaplan–Meier analysis did not show a significant difference in the event-free survival rate among the NLR tertiles (*P* < 0.775, Fig. 2). The results of univariable Cox regression analysis showed that age, systolic blood pressure, diastolic blood pressure, red blood cell count, left main coronary stenosis, stenosis of the right coronary artery, and high GS were predictors of MACEs (Table 5, all *P* < 0.05). Kaplan–Meier analysis also demonstrated that the risks of MACEs differed significantly different among the groups of different GS tertiles (*P* < 0.001). Multivariate Cox-regression analysis showed that age (HR: 1.049, 95% confidence interval [CI]: 1.024–1.075, *P* < 0.001), systolic blood pressure (HR: 1.029, 95% CI: 1.009–1.049, *P* = 0.005), and tertile of GS (3rd tertile vs. 1st tertile, HR: 3.216, 95% CI: 1.458–7.093, *P* = 0.004) were independent risk factors for MACEs.

**Table 5:** Predictors of the occurrence of MACEs in patients with new-onset ACS: results of univariate and multivariate Cox-regression analysesss

Variables	Univariate			Multivariate		
	HR	95% CI	*P*	HR	95% CI	*P*
Sex (Male/Female)	1.275	0.694–2.342	0.433			
Age	1.051	1.029–1.073	< 0.001	1.049	1.024–1.075	< 0.001
Hypertension	0.918	0.562–1.499	0.731			
Diabetes mellitus	1.175	0.676–2.044	0.568			
Smoking						
Former smoker vs never smoker	1.177	0.161–8.616	0.872			
Current smoker vs never smoker	0.842	0.516–1.374	0.490			
Alcohol drinking						
Former smoker vs never drinking	1.061	0.526–2.139	0.868			
Current smoker vs never drinking	1.390	0.797–2.423	0.246			
Family history of CAD	0.876	0.529–1.450	0.605			
SBP (mmHg)	1.029	1.016–1.042	< 0.001	1.029	1.009–1.049	0.005
DBP (mmHg)	1.040	1.019–1.059	< 0.001	1.007	0.978–1.038	0.626
Heart rate	1.008	0.992–1.024	0.319			
BMI	0.965	0.928–1.003	0.072			
HDL-C (mmol/l)	2.371	0.909–6.183	0.078			
LDL-C (mmol/l)	1.238	0.913–1.680	0.169			
TC (mmol/l)	1.132	0.905–1.416	0.278			
TG (mmol/l)	0.956	0.813–1.123	0.583			
ApoA1 (g/L)	1.170	0.662–2.067	0.589			
ApoB (g/L)	1.118	0.574–2.181	0.742			
Lp(a) (g/L)	0.999	0.997–1.001	0.150			
Creatinine (mmol/L)	1.001	0.994–1.006	0.995			
BUN (mmol/l)	0.978	0.881–1.086	0.675			
Uric acid (μmol/L)	1.001	0.997–1.003	0.980			
NLR group						
2nd tertile vs 1st tertile	1.155	0.643–2.075	0.629			
3rd tertile vs 1st tertile	0.940	0.509–1.734	0.843			
Monocyte count	0.923	0.427–1.996	0.839			
PLR	1.001	0.999–1.002	0.080			
RBC	1.534	1.015–2.318	0.042	1.452	0.991–1.038	0.056
HGB	1.002	0.993–1.012	0.660			
PLT	1.001	0.999–1.002	0.550			
MPV	1.001	0.847–1.180	0.997			
PDW	0.946	0.882–1.016	0.126			
PCT	1.654	0.177–15.465	0.659			
WBC	0.979	0.913–1.049	0.540			
Clinical diagnosis						
NSTEMI	1.062	0.386–2.9222	0.907			
STEMI	0.862	0.3692.012	0.731			
UPLMT	2.446	1.301–4.599	0.006	1.466	0.763–2.816	0.251
LAD	2.155	0.783–5.929	0.137			
LCX	1.430	0.836–2.445	0.191			
RCA	2.186	1.080–4.423	0.030	1.242	0.598–2.579	0.561
Aspirin	1.085	0.495–2.377	0.838			
Statins	1.230	0.562–2.695	0.605			
β-Blockers	0.756	0.237–2.410	0.637			
ACEI/ARB	0.813	0.199–3.323	0.773			
CCB	0.866	0.413–1.815	0.703			
PCI	0.614	0.280–1.345	0.223			
Gensini group						
2nd tertile vs 1st tertile	2.631	1.159–5.973	0.021	1.989	0.861–4.596	0.107
3rd tertile vs 1st tertile	5.076	2.363–10.900	< 0.001	3.216	1.458–7.093	0.004

## Discussion

The results of this retrospective cohort study showed that, although a higher NLR at baseline was independently associated with the severity of coronary lesions in new-onset ACS patients as evidenced by GS, the NLR was not a predictor of adverse clinical outcome during follow-up. We found that advanced age, elevated systolic BP, and higher GS are potential independent predictors of poor outcomes. Taken together, our results do not support incorporation of baseline NLR as a prognostic factor for new-onset ACS patients.

The key pathophysiologic processes for ACS include the rupture of a vulnerable plaque and subsequent formation of thrombosis [25, 26], and the role of inflammation in these processes has not only been confirmed by pathological studies, but also shown in some optical coherence tomography-based studies [6, 27]. Therefore, it has been proposed that the NLR, a novel but easily obtained marker of inflammation, may be a prognostic factor for ACS patients. Indeed, some previous studies suggested a prognostic role for the NLR in CAD patients. In a recent study with 636 STEMI patients, the NLR was significantly associated with in-hospital mortality [28]. Moreover, a post-hoc analysis showed that the NLR is associated with increased long-term mortality in patients with acute myocardial infarction (AMI) complicated by left main- and/or three-vessel disease [15]. However, in our retrospective cohort study, we did not find a significant association between a high NLR and poor prognosis in these patients, despite the relatively longer follow-up duration in our study compared with previous studies. The mechanisms have yet to be fully determined. Previous studies showed that the NLR changes dramatically, with the maximal level seen during the occurrence of inflammatory-related events [29]. Because neutrophils have a short life span and faster turnover, it is better to observe neutrophils in a dynamic manner rather than in a single measurement. Moreover, our study had a longer follow-up duration than previous ones, which may indicate that the potential prognostic role of the NLR in ACS is only acute. The relationships of NLR with ACS, overall mortality, and cancer survival have generally been thought to be driven by chronic inflammation [1, 2]. However, patients with a previous diagnosis of CAD were excluded in our study, and whether the NLR is associated with new-onset ACS has not been well established and remains incompletely understood. To the best of our knowledge, the potential link between NLR and new onset ACS has not been reported.

Another explanation is that the potential prognostic role of the NLR in ACS is confounded by factors related to the severity of coronary lesions, such as the GS. Therefore, the prognostic efficacy of the NLR is limited in a model that incorporates factors reflecting the severity of coronary lesions. Our results indicated that the NLR is significantly correlated with coronary lesion severity as evidenced by the GS. The results of our present study confirm the previous concept that inflammation correlates with the degree of coronary stenosis in CAD patients. Pathophysiologically, myocardial ischemia can induce an immediate rise in the plasma NLR, the magnitude of which is proportional to the severity of ischemia, although the neutrophil half-life is short [30]. Subsequently, a state of stress and inflammation, as seen in ACS patients, could result in increased levels of inflammatory markers in the blood circulation, accompanied by increased blood cortisol levels. An increase in cortisol has been shown to induce apoptosis, which in turn leads to lymphopenia and even inversion of the CD4 + /CD8 + T lymphocyte ratio [31]. Therefore, an elevated NLR represents an exaggerated inflammatory response that may reflect coronary atherosclerosis progression [16–18], and to some extent, may predict the acute prognosis in these patients [15, 32–34]. On the other hand, medications such as statins are well known to have anti-inflammatory actions [35], and the common use of statins during the post-acute phase of ACS may also reduce the prognostic efficacy of the NLR for long-term outcomes in ACS patients. Xinjiang is characterized by the integration of diverse ethnic cultures, but people in Xinjiang generally do not have a deep understanding of cardiovascular disease. Accordingly, a low treatment rate and poor adherence are common problems of hypertension management in this area. Therefore, it appears that although approximately 50% of the patients had hypertension, only 3–15% of patients were receiving treatment with antihypertensive agents. We are working hard to actively promote popularization of the science of cardiovascular diseases in different forms and languages in this region.

### Study limitations

First, as a retrospective observational single-center study with a small sample size, our study may be confounded by recall bias. Our results should be validated in prospective studies. Second, our study included consecutive patients with an initial diagnosis of ACS, and the diagnosis of the patients varied. Third, the NLR was only measured once at admission, and whether changes in the NLR during hospitalization or the NLR at discharge have an impact on the prognosis of these patients remains unknown. Finally, as the study was conducted over 8 years, PCI techniques and medical therapies likely evolved and changed with increasing evidence, which is likely to impact the outcome.

## Conclusions

The NLR may be associated with coronary lesion severity at baseline but is not associated with adverse outcomes in patients with new-onset ACS.

## Data Availability

The data that support the findings of this study are available from the Xinjiang Medical University Affiliated Hospital of Traditional Chinese Medicine. But restrictions apply to the availability of these data, which were used under license for the current study, and so are not publicly available. Data are however available from the authors upon reasonable request and with permission of the Xinjiang Medical University Affiliated Hospital of Traditional Chinese Medicine.

## Abbreviations

BMI: Body mass index;
SBP: Systolic blood pressure
DBP: Diastolic blood pressure
NLR: Neutrophil to lymphocyte ratio
WBC: White blood count
PLT: Platelet count
MPV: Mean platelet volume
PCT: Thrombocytocrit
PDW: Platelet distribution width
RBC: Red blood cell
HGB: Hemoglobin
BUN: Blood urea nitrogen
Cr: Creatinine
TC: Total cholesterol
TG: Triglyceride
HDL-c: High-density lipoprotein cholesterol
LDL-c: Low-density lipoprotein-cholesterol
Apo-AI: Apolipoprotein A1
Apo-B: Apolipoprotein B
Lp(a): Lipoprotein (a)
CCB: Calcium channel blocker
ACEI: Angiotensin-converting enzyme inhibitor
ARB: Angiotensin receptor blocker
CAD: Coronary artery disease
LAD: Left anterior descending artery
LCX: Left circumflex artery
RCA: Right coronary artery
UA: Unstable angina
NSTEMI: Non–ST-segment elevation myocardial infarction
STEMI: ST-segment elevation myocardial infarction
ACM: All-cause mortality
CM: Cardiac mortality
MACE: Major adverse cardiovascular events
ST: Stent thrombosis
PCI: Percutaneous transluminal coronary intervention
CI: Confidence interval
HR: Hazard ratio
